# Plasma cytokines for predicting diabetic retinopathy among type 2 diabetic patients via machine learning algorithms

**DOI:** 10.18632/aging.202168

**Published:** 2020-12-11

**Authors:** Bin Cao, Ning Zhang, Yuanyuan Zhang, Ying Fu, Dong Zhao

**Affiliations:** 1 Center for Endocrine Metabolism and Immune Diseases, Beijing Luhe Hospital, Capital Medical University, Beijing 101149, China; 2 Beijing Key Laboratory of Diabetes Research and Care, Beijing 101149, China

**Keywords:** plasma cytokines, diabetic retinopathy, machine learning algorithms, type 2 diabetes mellitus, prediction model

## Abstract

Aims: This study aimed to investigate changes of plasma cytokines and to develop machine learning classifiers for predicting non-proliferative diabetic retinopathy among type 2 diabetes mellitus patients.

Results: There were 12 plasma cytokines significantly higher in the non-proliferative diabetic retinopathy group in the pilot cohort. The validation cohort showed that angiopoietin 1, platelet-derived growth factor-BB, tissue inhibitors of metalloproteinase 2 and vascular endothelial growth factor receptor 2 were significantly higher in the NPDR group. Machine learning algorithms using the random forest yielded the best performance, with sensitivity of 92.3%, specificity of 75%, PPV of 82.8%, NPV of 88.2% and area under the curve of 0.84.

Conclusions: Plasma angiopoietin 1, platelet-derived growth factor-BB, and vascular endothelial growth factor receptor 2 were associated with presence of non-proliferative diabetic retinopathy and may be good biomarkers that play important roles in pathophysiology of diabetic retinopathy.

Materials and Methods: In pilot cohort, 60 plasma cytokines were simultaneously measured. In validation cohort, angiopoietin 1, CXC-chemokine ligand 16, platelet-derived growth factor-BB, tissue inhibitors of metalloproteinase 1, tissue inhibitors of metalloproteinase 2, and vascular endothelial growth factor receptor 2 were validated using ELISA kits. Machine learning algorithms were developed to build a prediction model for non-proliferative diabetic retinopathy.

## INTRODUCTION

Diabetic retinopathy (DR), one of the most prominent microvascular complications of diabetes mellitus (DM), is the leading cause of vision impairment and new-onset blindness in the working-age population and diabetes mellitus patients [1, 2]. The increase in the global prevalence of diabetic eye diseases, comprising DR and diabetic macular edema (DME), is intimately connected to the soaring prevalence of DM [3–5]. It was reported that across China, the prevalence of DR and sight-threatening DR were 27.9% and 12.6% in diabetic patients, respectively [6].

For algorithm development, deep learning techniques have been used for automated detection of DR and DME, based on features in retinal fundus photographs and achieved robust performance [7–10]. Although image-based features of DR are well-known, knowledge about its protein phenotype are limited. It is accepted that angiogenesis and inflammation crosstalk are intrinsic components of DR [11, 12]. Increasing evidence shows that, in retinal cells and tissues, various cytokines, including vascular endothelial growth factor (VEGF), matrix metalloproteinases (MMPs), and tissue inhibitors of metalloproteases (TIMPs), play essential roles in the progress of DR via angiogenic, inflammatory and fibrotic reactions [13–17]. Thus, cytokines play important roles in the pathophysiology of DR. However, the associations between plasma cytokines and non-progressive DR (NPDR) are unclear.

This is the first study to investigate the associations between plasma cytokines and non-progressive DR (NPDR) and to build a prediction model for NPDR. In this study, we hypothesized that the pathological processes leading to NPDR caused characteristic changes in the concentrations of plasma proteins. We then investigated the characteristic changes in plasma cytokines, generating a detectable disease-specific protein phenotype, and finally developed machine learning classifiers for NPDR at the protein level.

## RESULTS

### Study subjects

For plasma protein profiling, 14 patients with NPDR and 14 patients with T2DM were selected as the pilot cohort. The mean ages of patients with NPDR or T2DM were 62.71 vs. 58.50 years, respectively, and the median durations of diabetes were 13.57 vs. 8.08 years, respectively. The proportion of hypertension was significantly higher in the NPDR group (78.6% vs. 28.6%, p = 0.023). For validation, 115 patients with NPDR and 115 patients with T2DM were selected as the validation cohort. The mean ages of patients with NPDR or T2DM were 60.40 vs. 58.63 years, respectively, and the median durations of diabetes were 8.69 vs. 6.92 years, respectively. In the same manner, the proportion of hypertension was significant higher in the NPDR group (60.9% vs. 47.0%, p = 0.047) (Table 1).

**Table 1:** Clinical characteristics of the study population.

**Clinical characteristics**	**Pilot cohort**				**Validation cohort**		
	**DM (n=14) (Mean ± SD)**	**DR (n=14) (Mean± SD)**	**p**		**DM (n=115) (Mean ± SD)**	**DR (n=115) (Mean ± SD)**	**p**
Age (years)	58.50±8.31	62.71±7.63	0.174		58.63±14.24	60.40±12.04	0.316
BMI (Kg/m^2^)	24.83±2.38	27.42±4.60	0.081		25.74±3.90	26.03±3.81	0.594
Duration of diabetes (years)	8.08±8.73	13.57±10.24	0.153		6.92±8.53	8.69±8.19	0.116
Fasting plasma glucose (mmol/L)	8.08±8.73	13.57±10.24	0.118		8.92 ±3.24	8.82 ±4.03	0.847
HbA1c (%)	9.36±2.28	9.59±1.55	0.766		9.85 ±2.13	9.31 ±2.14	0.060
Fasting C peptide (mIU/L)	1.49±0.59	1.68±1.04	0.569		1.53 ±1.00	1.76 ±1.05	0.111
2-h post prandial C-peptide (mIU/L)	5.19±3.86	3.90±2.21	0.320		3.74 ± 2.70	3.96 ± 2.32	0.529
Triglyceride (mmol/L)	2.05±1.54	1.93±1.27	0.836		1.80 ± 1.39	1.78 ±1.08	0.925
Total cholesterol (mmol/L)	4.85±2.29	4.94±1.18	0.917		4.46 ± 1.29	4.45 ±1.08	0.947
Low-density lipoprotein (mmol/L)	3.08±1.65	3.11±0.78	0.955		2.85 ± 1.00	2.86 ± 0.85	0.949
Gender, male (%)	8 (57.1%)	4 (28.6%)	0.252		62 (53.9%)	44 (38.3%)	0.025
Hypertension, number (%)	4 (28.6%)	11 (78.6%)	0.023		54 (47.0%)	70 (60.9%)	0.047
*Diabetic nephropathy, number (%)	2 (14.3%)	4 (28.6%)	0.645		34 (31.8%)	46 (41.1%)	0.198
Diabetic peripheral neuropathy, number (%)	0 (0%)	0 (0%)	1		2 (11.7%)	1 (0.9%)	1
Diabetic foot, number (%)	0 (0%)	0 (0%)	1		0 (0%)	0 (0%)	1

*, 11 missing data in validation cohort.

### Identification of predominant plasma cytokines in NPDR patients

**Figure 1:**
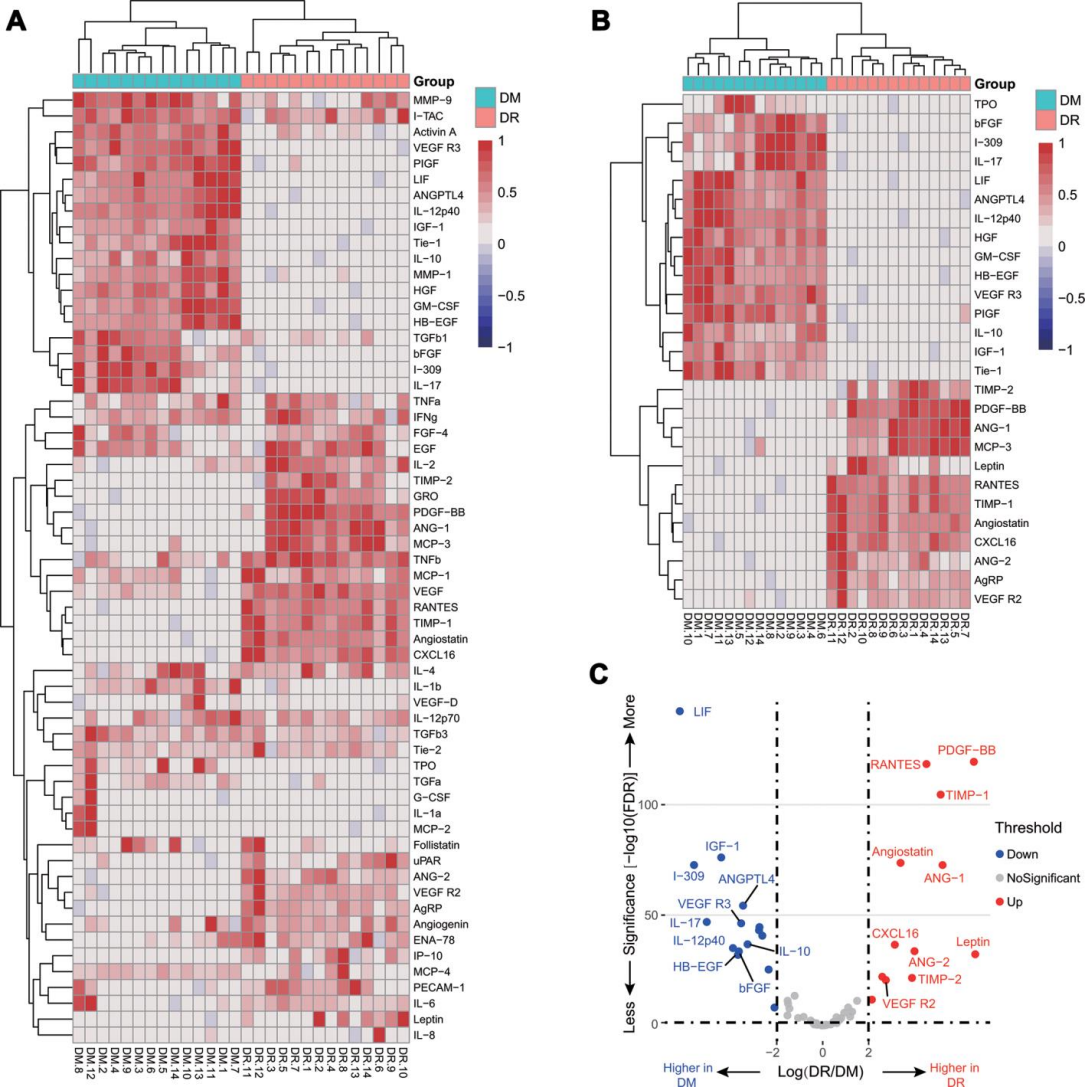
**Relative cytokine changes in the pilot cohort.** A heat map of relative changes of all 60 plasma cytokines (**A**); a heat map of 27 cytokines with a fold change larger than 4 or less than 0.25 (**B**); a volcano plot of the top 10 increasing and decreasing cytokines (**C**).

We profiled plasma cytokines by using the human glass-based arrays and obtained semi-quantifiable results for 60 plasma cytokines. Compared with T2DM patients, the relative changes of the 60 cytokines were shown in Figure 1A. There were 27 cytokines significantly different between the two groups, among which the fold change of 12 plasma cytokines were larger than four (Figure 1B). As shown in the volcano plot, the top 10 increased cytokines were PDGF-BB, leptin, ANG-1, TIMP-1, RANTES, TIMP-2, ENA-78, angiostatin, CXCL16, and VEGFR2, and the top 10 decreased cytokines were IL-10, ANGPTL4, bFGF, VEGFR3, HB-EGF, IL-12p40, IGF-1, IL-17, I-309, and LIF (Figure 1C). Based on the top 10 increased cytokines, PCA was performed, showing a clear separation between the two groups (Supplementary Figure 1). These findings suggested that plasma cytokines may be helpful to distinguish NPDR from T2DM patients.

### Validation of the six increased plasma cytokines in a large-scale cohort

**Figure 2:**
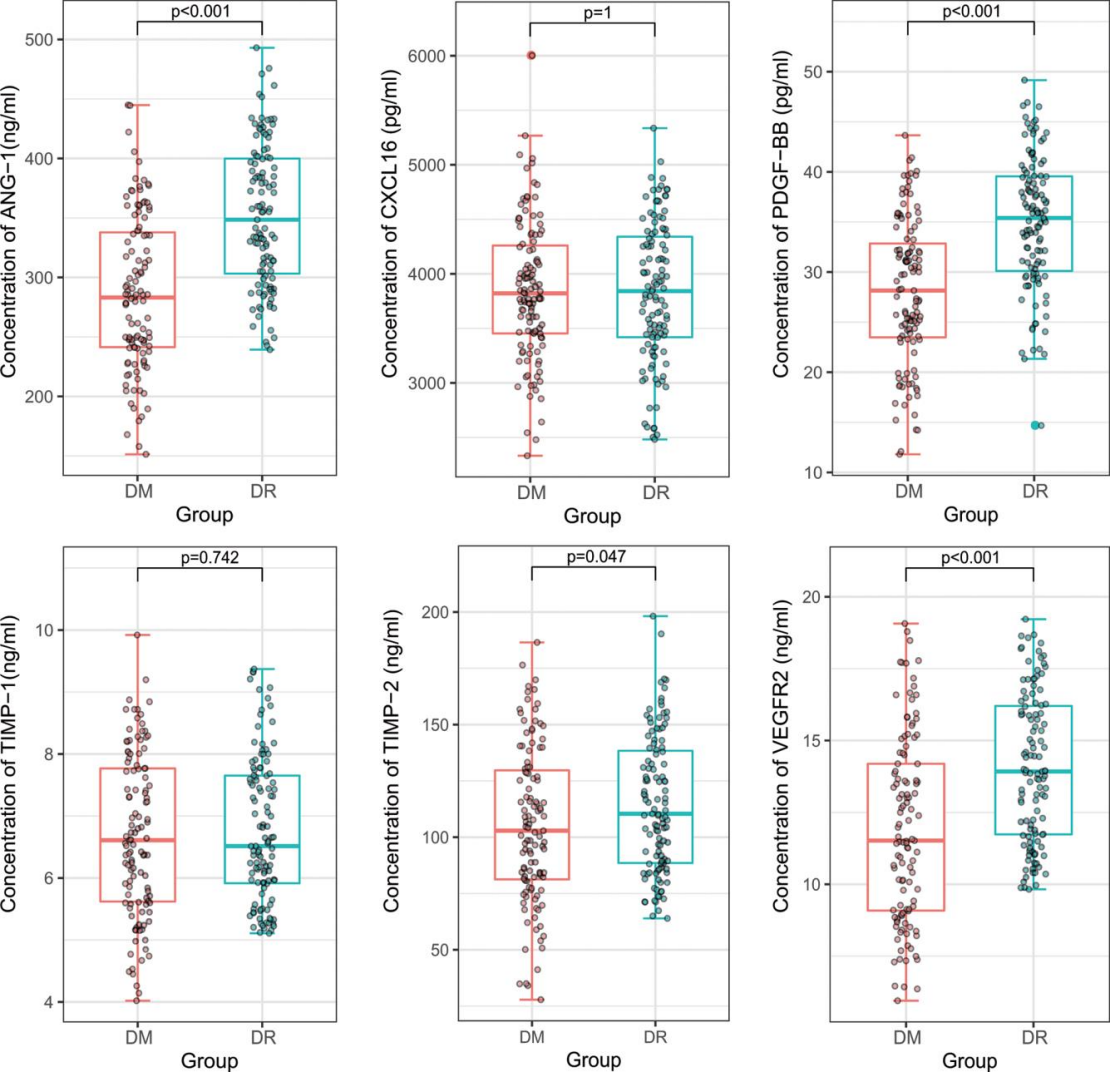
**A comparison of plasma concentrations of PDGF-BB, TIMP-1, TIMP-2, ANG-1, CXCL16, and VEGFR2 in the validation cohort.** ANG-1, PDGF-BB, TIMP-2, and VEGFR2 were significantly higher in non-proliferative diabetic retinopathy patients than in diabetes mellitus patients. However, there were no significant difference of CXCL16 and TIMP-1.

We further measured the plasma concentration of PDGF-BB, TIMP-1, TIMP-2, ANG-1, CXCL16, and VEGFR2 by ELISA kits in a large cohort, which comprised 115 NPDR and 115 T2DM patients. The concentrations of ANG-1, PDGF-BB, TIMP-2, and VEGFR2 (351.85 ng/mL, 34.95 pg/mL, 114.60 ng/mL, and 14.06 ng/mL, respectively) were significantly higher in NPDR samples than those in T2DM patients (286.81 ng/mL, 28.07 pg/mL, 105.01 ng/mL, and 11.91 ng/mL, respectively). However, there was no significant difference of CXCL16 and TIMP-1 (3,828.94 vs. 3,849.86 pg/mL and 6.78 vs 6.68 ng/mL, respectively) (Figure 2).

### Correlation between cytokines and clinical characteristics

Pearson’s correlation analysis was performed to investigate the potential relationships among cytokines and clinical characteristics. For NPDR, PDGF-BB was weakly correlated with diabetic duration (r = 0.34), and VEGFR2 was weakly correlated with total cholesterol (r = 0.33) and low-density lipoprotein (r = 0.30) (Supplementary Figure 2A). For T2DM patients, there was no obvious relationship between plasma cytokines and clinical characteristics (Supplementary Figure 2B).

The proportion of hypertension was significant higher in the NPDR group in both the pilot and validation cohorts. To further eliminate the interference of hypertension on the six plasma cytokines, we focused on comparing concentrations of the six plasma cytokines in patients with or without hypertension. Supplementary Figure 3 shows that there was no significant difference of the mean levels of ANG-1, CXCL16, PDGF-BB, TIMP-1, TIMP-2, and VEGFR2 in the NPDR and T2DM groups (322.83 vs. 315.24 ng/mL, 3,796.44 vs. 3,889.65 pg/mL, 32.17 vs. 30.74 pg/mL, 6.70 vs. 6.77 ng/mL, 107.13 vs 112.94 ng/mL, and 12.99 vs. 12.99 ng/mL, respectively). Thus, the higher concentration of these six cytokines in NPDR patients may have minimal association with hypertension in this study.

### Feature selection for the machine learning algorithms

We then used PCA to compute the relative contributions of each cytokine to the separation among NPDR and T2DM patients. The first and second principal components of the PCA plot (Dim1 and Dim2) accounted for 36.0%, and 17.1% of the variations, respectively, in the dataset. The projections of samples in PCA were distinguished with relatively small overlapping areas. CXCL16 and TIMP-1 contributed more to the second principal component than the first principal component, while ANG-1, PDGF-BB, TIMP-2, and VEGFR2 contributed more to the first principal component (Supplementary Figure 4A). To be specific, the contribution order of cytokines to the first principal component were ANG-1 (25.9%), PDGF-BB (21.0%), TIMP-2 (20.2%), VEGFR2 (16.5%), TIMP-1 (9.9%), and CXCL16 (6.5%) (Supplementary Figure 4B).

Random forest was performed to evaluate the importance level of each cytokine to the separation among NPDR and T2DM patients. The importance level of ANG-1 (22.8%), VEGFR2 (22.2%), and PDGF-BB (20.5%) were higher than TIMP-1 (13.7%), TIMP-2 (10.5%), and CXCL16 (10.3%) (Supplementary Figure 4C).

Lasso regression was also conducted for model selection. The coefficients of ANG-1, VEGFR2, and PDGF-BB in lasso regression were 0.014, 0.003 and 0.001, while the coefficient of TIMP-1, TIMP-2, and CXCL16 were 0 (Supplementary Figure 4D).

Finally, combined PCA, random forest, and lasso regression results for ANG-1, VEGFR2, and PDGF-BB were selected for a machine learning prediction model building.

### Development and validation of machine learning classifiers

**Figure 3:**
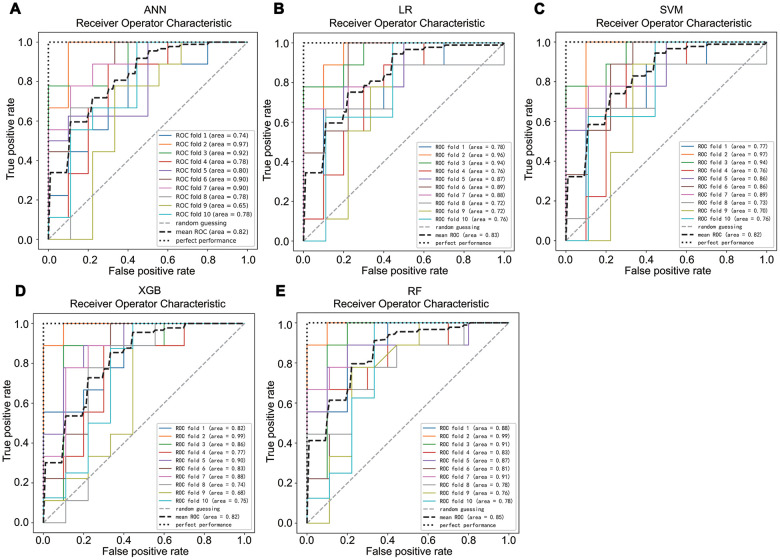
****The average area under the curve of a 10-fold cross validation of ANN (**A**), LR (**B**), SVM (**C**), XBG (**D**), and RF (**E**) in the train set.

To select a high-performance classifier for prediction, we developed ANN, LR, SVM, XBG, and RF classifiers based on ANG-1, VEGFR2, and PDGF-BB. In 10-fold cross validation of the train set, the mean AUC of ANN, LR, SVM, XBG, and RF were 0.82, 0.83, 0.82, 0.82, and 0.85, respectively (Figure 3). This finding revealed that all classifiers performed similarly and exhibited excellent performance in the training set.

For validation, the test set was used to evaluate the performance of machine learning classifiers. Table 2 and Supplementary Table 1 show the comparison results of machine learning algorithms in the test set. LR, ANN, and SVM exhibited moderate predictive performance, with accuracy ranging from 72% to 76%, sensitivity ranging from 73.1% to 84.6%, specificity ranging from 65% to 70%, AUCs ranging from 0.72 to 0.75, and f1 scores ranging from 0.75 to 0.80. XGB exhibited good predictive performance, with an AUC of 0.82 and an f1 score of 0.85. RF classifier performed best in all the following indicators; the accuracy was 85%, the sensitivity was 92.3%, the specificity was 75%, the PPV was 82.8%, the NPV was 88.2%, the AUC was 0.84, and the f1 score was 0.87, while LR performed worst. The McNamara’s test was conducted to statistically compare performance of RF (the best model) and LR (the worst model) and p value equal to 0.289. Combined these results, we can calculate that, RF was the best classifier, although there was no statistical difference compared with other models.

**Table 2:** Performance of the 5 machine learning classifiers on the test set.

	**Model**	**Accuracy**	**Sensitivity**	**Specificity**	**AUC**	**F1 score**
**Test set**	LR	72%	73.1%	70.0%	0.72	0.75
	ANN	72%	80.8%	70.0%	0.75	0.83
	SVM	76%	84.6%	65.0%	0.75	0.80
	XGB	83%	88.5%	75.0%	0.82	0.85
	RF	85%	92.3%	75.0%	0.84	0.87

## DISCUSSION

Because of the main role that angiogenesis and inflammation have in the development and progression of NPDR, we hypothesized that angiogenesis- and inflammation-related cytokines in the plasma might be different in NPDR patients, and could be novel predictive biomarkers. To the best of our knowledge, this is the first large-scale study to determine specific plasma cytokines for the diagnosis of NPDR when compared with those in T2DM patients. In the pilot cohort with a small number of samples, cytokines antibody arrays were performed to identify 60 plasma cytokines. The results showed that 27 cytokines were increased in patients with NPDR, among which 12 cytokines were increased in the NPDR group (fold change > 4). In the larger-scale validation cohort, ELISA kits were used to validate six of the 12 plasma cytokines. Four out of six plasma cytokines, ANG-1, PDGF-BB, TIMP-2, and VEGFR2, were confirmed to be significantly higher in NPDR patients. These results suggested that plasma cytokines may be specifically involved in the development of NPDR.

The main goal of this study was to identify potential plasma biomarkers of patients with NPDR. Feature selection indicated that NPDR was highly associated with ANG-1, PDGF-BB, and VEGFR2, so these three cytokines were finally included in the machine learning algorithms. LR, ANN, SVM, RF, and XGB classifier confirmed that these three cytokines were highly discriminatory for NPDR in the independent test set, with the sensitivity ranging from 73.1% to 92.3%, with the specificity ranging from 65.0% to 75%, and with the AUC ranging 0.72 to 0.84. Among the five machine learning algorithms, RF classifier, with a sensitivity of 92.3% and the AUC of 0.84 in the test set, showed excellent discrimination of NPDR from T2DM patients.

Angiogenesis- and inflammation-related cytokines play vital roles in injuries of human retinal endothelial cells in culture. ANG-1, a member of the angiopoietins family, is a growth factor that plays a key role in vessel homeostasis, angiogenesis, and vascular permeability via interacting with the Tie2 transmembrane receptor tyrosine kinase [18–20]. Damage of the blood retinal barrier, which is induced by diabetes, is inhibited by Ang-1 in a dose-dependent manner [21]. PDGF-BB has been reported to be involved in astrogliosis and the formation of proliferative membranes in retinopathy by activating PDGFRα and PDGFRβ [22]. The upregulated combination of VEGF-A and VEGFR2 is a response to the ischemia induced by retinal vascular damage, and stimulates extraretinal vascular outgrowth to the retinal surface without amelioration of ischemia in the retina [23–25]. TIMP-2 is an endogenous inhibitor of matrix metalloproteinase-2 and may act as a protector to reduce the loss of capillary cells resulting in the development of diabetic retinopathy [26, 27]. Although angiogenesis- and inflammation-related cytokines are involved in the development and progression of DR, their changes in DR are unclear.

With the development of an algorithm, deep learning techniques have been used for automated detection of DR. Based on features in retinal fundus photographs, deep learning algorithms show discriminative abilities comparable with those of ophthalmologists [7, 8, 28–31]. Image features of DR are well-known; however, knowledge about its plasma protein specific features are limited. In the present study, the RF classifier, which was based on the plasma concentrations of ANG-1, PDGF-BB, and VEGFR2, also showed good prediction abilities. Although the performance of the plasma protein-based RF classifier was not as good as that of the image-based deep learning classifier, our results indicated that plasma ANG-1, PDGF-BB, and VEGFR2 may be protein specific features of NPDR, and the roles of these three plasma cytokines in the pathophysiology of NPDR, are worthy of further study.

Two previous studies reported that serum levels of ANG-1 were significantly higher in the NPDR group, when compared to the T2DM group [32, 33]. Paine et al. also reported that the plasma levels of soluble VEGFR2 consistently increased with the severity of DR [34]. Consistent with these findings, in the present study, we showed that in NPDR, ANG-1, TIMP-2, VEGFR2, and PDGF-BB were significantly increased. The protective cytokines, ANG-1 and TIMP-2, were increased in the NPDR group. A possible explanation for this might be that the increases of ANG-1 and TIMP-2 may represent an adaptive compensatory mechanism to promote cellular repair, to suppress the development of retinal or choroidal neovascularization, and to strengthen the integrity of the vascular structure [35].

The plasma cytokine changes in NPDR patients have been controversial, and the correlations between plasma cytokines and clinical features were also unclear. Pearson’s correlation indicated that for NPDR patients, PDGF-BB was weakly correlated with the duration of diabetes (r = 0.34), and VEGFR2 was weakly correlated with total cholesterol (r = 0.33) and low-density lipoprotein (r = 0.30). According to previous studies, diabetic duration, total cholesterol, and low-density lipoprotein were risk factors for diabetic retinopathy [6, 36, 37]. Whether PDGF-BB and VEGFR2 act independently or in concert with blood lipids during NPDR is still unclear, so further studies are needed.

The strengths of this study were as follows. It was the first study to include a large number of patients with comparable baselines. It contained a pilot study for screening of possible cytokines associated with NPDR and a large-scale cohort for ELISA verification Machine learning algorithms based on these plasma cytokines exhibited good performance for distinguishing DR from T2DM patients. However, we acknowledge several limitations in our study. First, the examination for DR was based on two-field fundus photographs, which are theoretically less sensitive than seven-field retinal photographs. However, the presence of mild DR would be underestimated only if the retinal pathologies were present in the peripheral area of the retina. Second, patients with coronary heart disease (CHD) were excluded in this study. The diagnosis of CHD, however, was based on the history of disease provided by the patients. A significant percentage of diabetic patients with coronary heart disease usually have no symptoms, so it may be inevitable that a few CHD patients were included in this study.

In summary, we report that plasma cytokines including ANG-1, PDGF-BB, TIMP-2, and VEGFR2 were increased, and that plasma cytokine patterns were comprehensive predicators of DR based on machine learning algorithms. Our results suggested that plasma cytokines could be strong risk markers of NPDR.

## MATERIALS AND METHODS

### Patients

Inpatient patients with NPDR or Type 2 DM (T2DM) were enrolled in this study at the Center for Endocrine Metabolism and Immune Diseases of Beijing Luhe Hospital, Capital Medical University (Beijing, China) between November 2018 and September 2019. Two different groups were established: (1) an age- and body mass index (BMI)-matched pilot cohort containing 14 NPDR patients and 14 T2DM patients to comprehensively screen changes of angiogenesis- and inflammation-related plasma cytokines by human glass-based cytokine microarrays. (2) A validation cohort containing 115 NPDR patients and 115 T2DM patients to further measure the concentrations of plasma angiopoietin 1 (ANG-1), CXC-chemokine ligand 16 (CXCL16), platelet-derived growth factor-BB (PDGF-BB), tissue inhibitors of metalloproteinase 1 (TIMP-1), tissue inhibitors of metalloproteinase 2 (TIMP-2), and vascular endothelial growth factor receptor 2 (VEGF R2) using ELISA kits.

The 2010 diagnostic criteria of T2DM from the American Diabetes Association were used: (1) glycosylated hemoglobin (HbA1c) ≥ 6.5%; (2) fasting plasma glucose ≥ 7.1 mmol/L; (3) 2 h of blood glucose during an oral glucose tolerance analysis ≥ 11.1 mmol/L; and (4) in a typical hyperglycemic or hyperglycemia crisis patient, random blood glucose was ≥ 11.1 mmol/L. Two-field retinal photographs were taken of each eye of all patients by a trained photographer, using a nonmydriatic fundus camera (Topcon, Tokyo, Japan). The diagnosis and grading of DR were conducted by two trained specialists following the Early Treatment of Diabetic Retinopathy Study Researched Group (ETDRS) [38] as follows: (1) no retinopathy; (2) mild NPDR; (3) moderate NPDR; (4) severe NPDR; and (5) proliferative retinopathy (PDR). Patients met the following inclusion criteria: (1) conformity to the above diabetes diagnostic criteria; (2) conformity to NPDR diagnostic criteria; and (3) > 18 years of age. The exclusion criteria were: (1) Type 1 DM or other type of DM; (2) any retinopathy other than NPDR; (3) acute complications of diabetes; and (4) a history of cardiovascular diseases and stroke.

### Clinical examination and data collection

Blood biochemical parameters, including fasting glucose, HbA1c, 2 h postprandial C-peptide, triglycerides, total cholesterol, and low-density lipoprotein were collected at the time of the screening. All information from the patients, including height, weight, diabetic related complications, other histories of diseases, retinal examination, and optical coherence tomography, were recorded. Plasma samples were collected in ethylenediaminetetraacetic acid tubes and were immediately centrifuged at 1,400 × g for 10 min at 4° C, and then the supernatant was aliquoted and stored at -80° C, avoiding freeze thaw cycles. All samples were collected with the signed informed consent from all patients, and all related procedures were performed with the approval of the internal review and ethics boards of the indicated hospitals.

### Cytokine antibody assay

Plasma soluble cytokines were measured in duplicate using the Ray Biotech G-Series Human Angiogenesis Array 2 and 3 (Ray Biotech, Norcross, GA, USA) following the recommended protocols. Briefly, all samples were biotinylated. The antibodies were immobilized in specific spot locations on glass slides. The incubation of array membranes with biological samples resulted in the binding of cytokines to the corresponding antibodies. Signals were visualized using streptavidin-horseradish peroxidase conjugates and colorimetric assays. Final spot intensities were measured as the original intensities after subtracting the background. The two kits provided high sensitivity and specificity to simultaneously detect a total of 60 cytokines from the plasma. As determined by densitometry, the inter-array coefficient of variation of spot signal intensities was less than 20%.

### Differential protein level analysis

To identify proteins with significant concentrations in the plasma, the raw data were normalized and then the fold change of NPDR vs. T2DM for each cytokine was calculated using the “edgeR” package [39]. The fold change values of cytokines were used to indicate their relative concentration levels. Any fold change ≥ 2 or ≤ 0.5 with FDR < 0.05 was considered as significant. Based on differential plasma protein, principle component analysis (PCA) was conducted to evaluate variation between two groups using the “ggbiplot” package.

### ELISA validation

Plasma concentration of PDGF-BB, TIMP-1, TIMP-2, ANG-1, CXCL16, and VEGFR2 were determined in the validation cohort by an ELISA kit following the manufacturer’s instructions (Human ELISA kit, MLbio, Shanghai, China). The intra-assay coefficient of variation was 10%, and the inter-assay coefficient of variation was 12%. No significant cross-reactivity or interference was observed.

### Machine learning algorithms to distinguish NPDR from T2DM patients

The whole data set was randomly divided into the training set (80%) and the test set (20%). To prevent overfitting, the training set was randomly split into 10 equal-sized subgroups using the 10-folds cross validation method. In 10-folds cross validation, nine subgroups were retained as training data and the remaining one subgroup was used as the validation data for testing the model. The cross-validation process was then repeated 10 times, with each of the 10 subsamples used exactly once as the validation data. The 10 results from the folds then were averaged to produce a single estimation. Finally, the test set was used to evaluate the model (Supplementary Figure 5). Five machine learning algorithms were trained: the artificial neural network (ANN), logistic regression (LR), random forest (RF), support vector machine (SVM), and xgradient-boosting (XGB). Parameters for each machine learning method were shown in Supplementary Table 2. The performance of each classifier was evaluated by its accuracy, sensitivity and specificity, positive predictive value (PPV), negative predictive value (NPV), f1 score, Matthews correlation coefficient (MCC), and area under the curve (AUC) of the receiver operating characteristic (ROC).

### Statistical analysis

Differences in clinical characteristics and cytokines between groups were calculated using the Wilcoxon test for continuous variables and the chi-square test for categorical variables. Pearson’s correlation was performed to assess the relationship of the plasma cytokines and clinicopathological characteristics. Machine learning algorithms and diagnostic performance were evaluated using scikit-learn (V0.21.3) in Python V3.7.4. Other data analysis and visualization were performed by R software, version 3.6.2 (The R Project for Statistical Computing, Vienna, Austria). A two-sided P-value < 0.05 was considered statistically significant.

## Supplementary Material

Supplementary Figures

Supplementary Tables
