# Supplementary material for: Plasma cytokines for predicting diabetic retinopathy among type 2 diabetic patients via machine learning algorithms
Source: Aging (Albany NY). 2020 Dec 11;13(2):1972–88. doi: 10.18632/aging.202168 (PMC7880388; doi:10.18632/aging.202168)
Supplement: Supplementary Figures [file aging-13-202168-s001.pdf]

SUPPLEMENTARY FIGURES

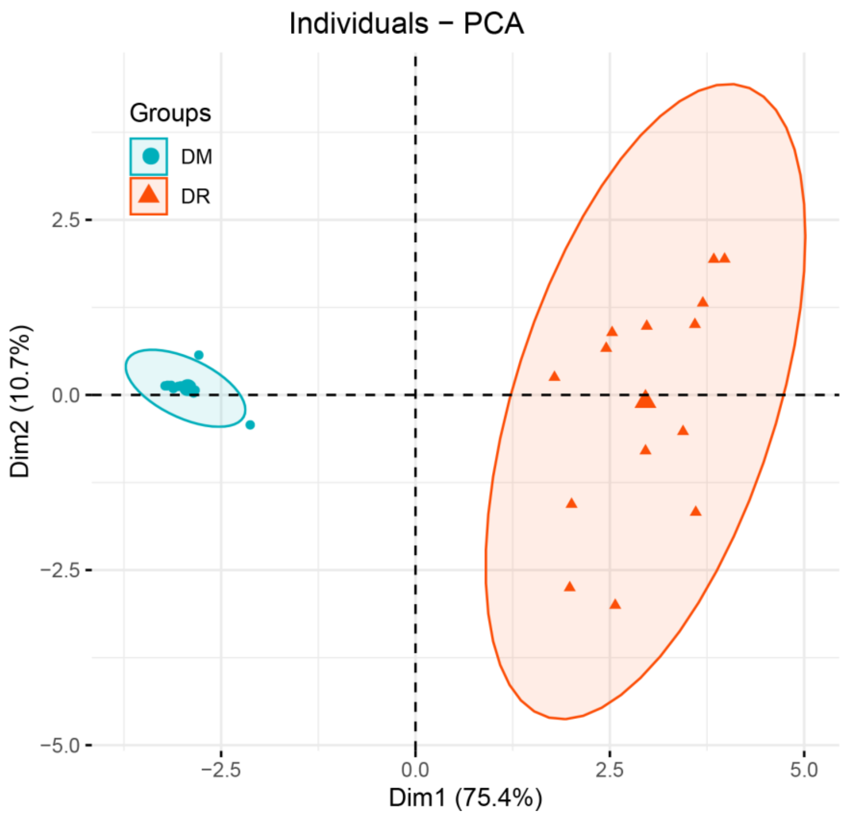

Supplementary Figure 1. Principle component analysis for the pilot cohort. There was a clear separation between the two groups.

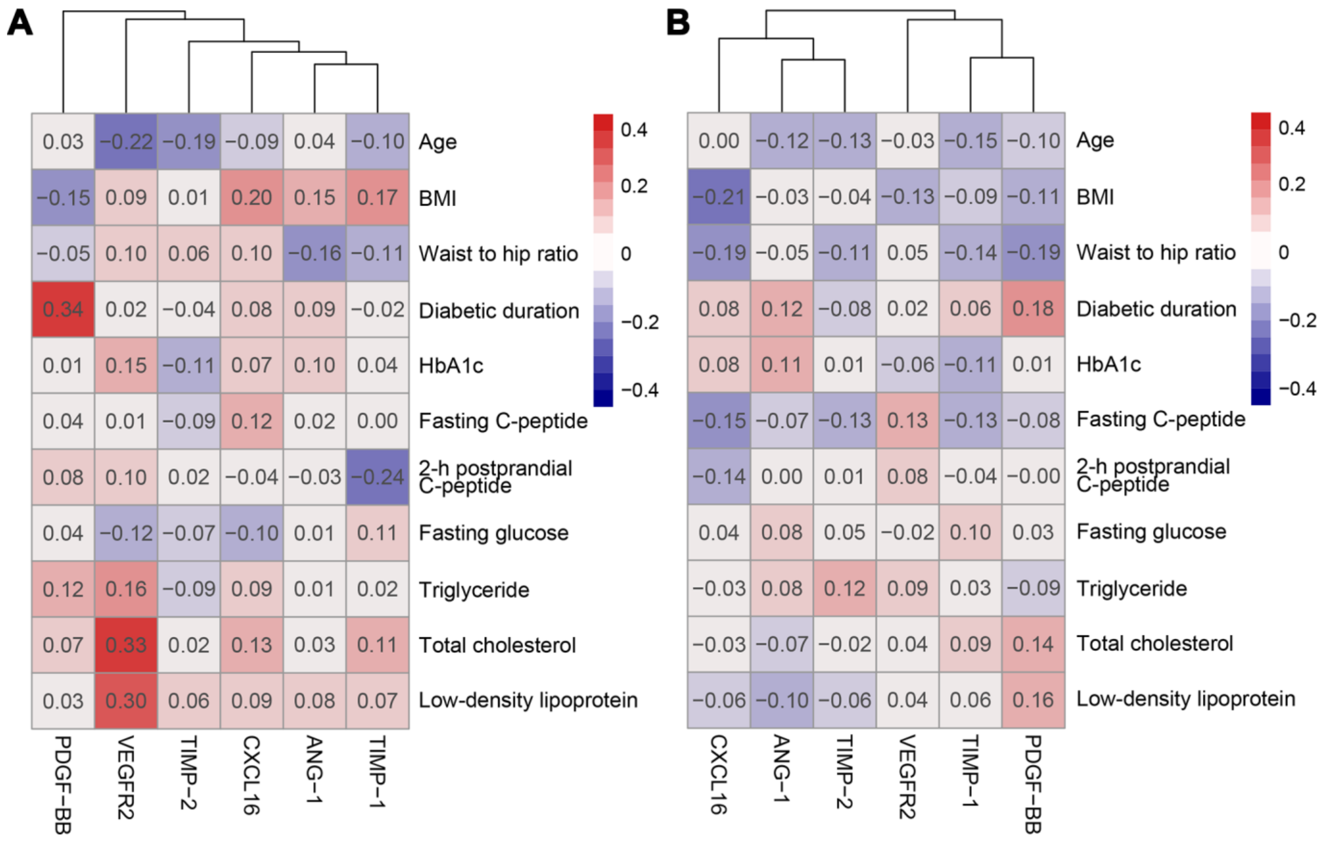

Supplementary Figure 2. Pearson's correlation of plasma cytokines and clinical characteristics in non-proliferative diabetic retinopathy patients (**A**) and diabetes mellitus patients (**B**).

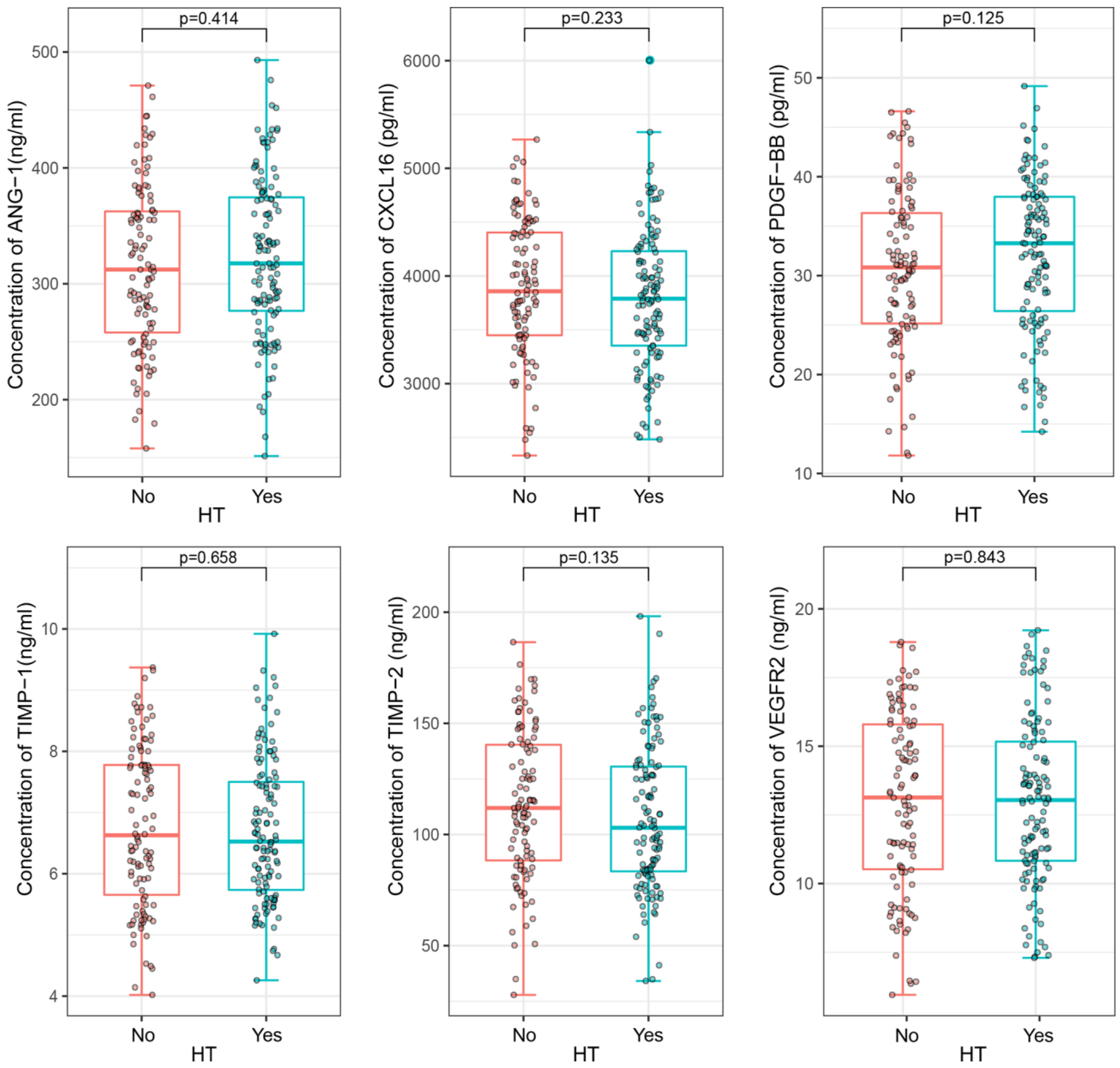

Supplementary Figure 3. Comparison of plasma concentrations of PDGF-BB, TIMP-1, TIMP-2, ANG-1, CXCL16, and VEGFR2 in patients with or without hypertension. There was no significant difference in the mean levels of ANG-1, CXCL16, PDGF-BB, TIMP-1, TIMP-2. and VEGFR2 in the diabetic retinopathy and diabetes mellitus groups.

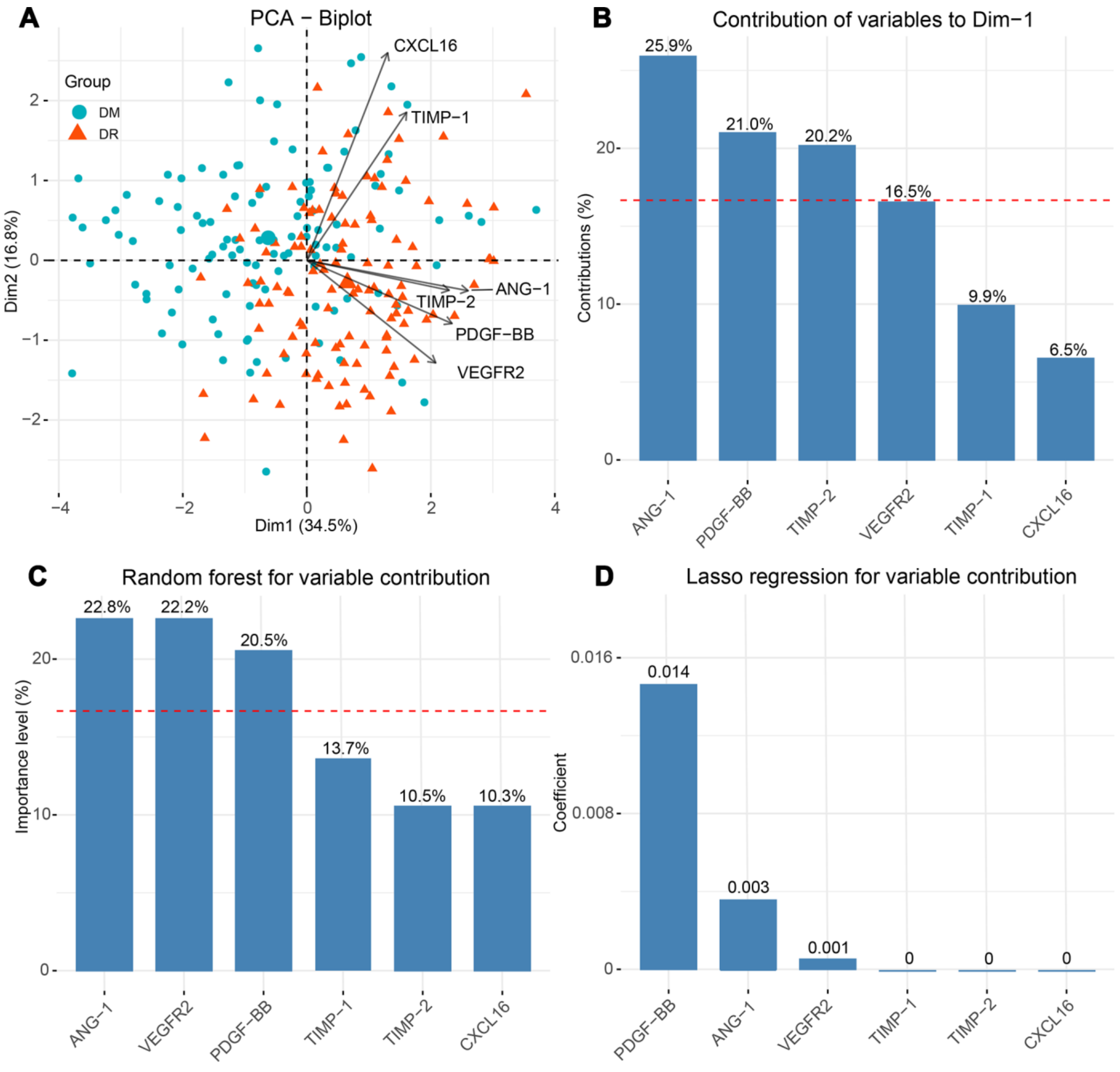

Supplementary Figure 4. Feature selection for machine learning algorithms. Principle component analysis to compute the relative contribution of each cytokine to the separation among the non-proliferative diabetic retinopathy and diabetes mellitus patients. (**A**); The contribution of each cytokine to the first principal component (**B**); use of the random forest to evaluate the importance level of each cytokine to the separation among diabetic retinopathy and diabetes mellitus patients (**C**). use of the Lasso regression for feature selection (**D**).

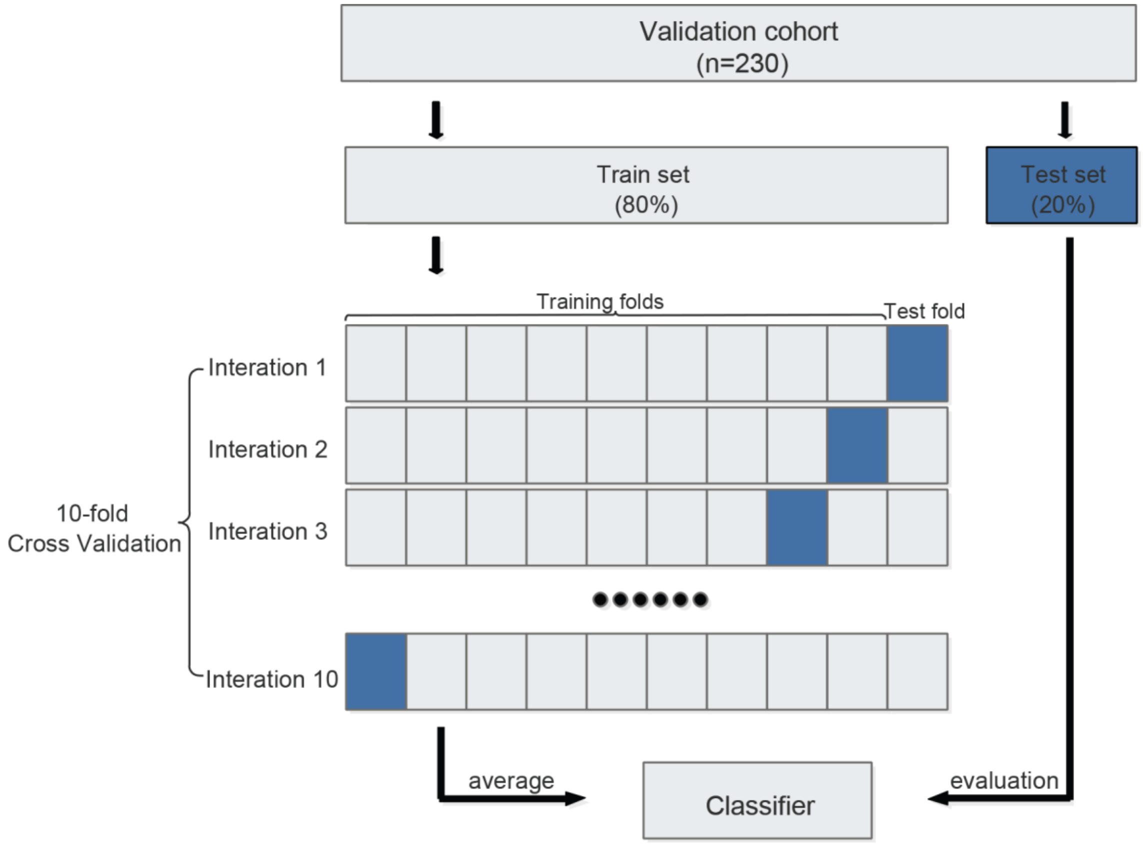

Supplementary Figure 5. Flow chart of the 10-fold cross validation method.
