# Supplementary material for: Plasma cytokines for predicting diabetic retinopathy among type 2 diabetic patients via machine learning algorithms
Source: Aging (Albany NY). 2020 Dec 11;13(2):1972–88. doi: 10.18632/aging.202168 (PMC7880388; doi:10.18632/aging.202168)
Supplement: Supplementary Tables [file aging-13-202168-s002.pdf]

## SUPPLEMENTARY TABLES

**Supplementary Table 1. Performance of the 5 machine learning classifiers on the test set.**

| | Model | PPV | NPV | MCC | Model processing time (second) |
|---|---|---|---|---|---|
| **Test set** | LR | 76.0% | 66.7% | 0.43 | 0.28 |
| | ANN | 77.8% | 73.7% | 0.43 | 1.80 |
| | SVM | 75.9% | 76.5% | 0.51 | 0.36 |
| | XGB | 82.1% | 83.3% | 0.64 | 0.99 |
| | RF | 82.8% | 88.2% | 0.69 | 0.96 |

**Supplementary Table 2. Parameters for each machine learning method in this study.**

| Model | Optimal parameters |
|---|---|
| LR | Penalty: ‘l2’, C: 0.1 |
| SVM | C: 0.1, gamma: 0.01, kernel: ‘rbf’ |
| RF | n estimators: 80, max depth: 8, min samples split: 3, min samples leaf: 1 |
| XGB | n estimators: 80, max depth:3, learning rate:0.1 |
